# Psychotherapeutic Intervention for Adults With Acquired Brain Injury: A Case Study Using BackUp

**DOI:** 10.3389/fresc.2022.771416

**Published:** 2022-02-18

**Authors:** Cecilie Marie S. Thøgersen, Chalotte Glintborg, Tia G. B. Hansen, Johan Trettvik

**Affiliations:** Department of Communication and Psychology, Center for Developmental and Applied Psychological Science, Aalborg University, Aalborg, Denmark

**Keywords:** pilot study, mixed method, single case, psychological intervention, acquired brain injury

## Abstract

A moderate-to-severe acquired brain injury (ABI) can have tremendous lifelong consequences for ABI-survivors and their families. Despite rehabilitation practice since the 1980s aspiring to a dynamic, coherent and holistic approach, the psychological dimension still seems to be a challenge and research has revealed persisting psychosocial impairments after ABI. Therefore, we developed BackUp©, a manual based short term psychological intervention for adults with ABI. This study explores the effect of the intervention though a small feasibility study, employing a single case design. One client received the intervention. Self-report measures were collected, and a semi structured interview was conducted. While results from pre, post and follow-up measures do not show clear positive results, the interview reveals positive experiences and the participant reported achieving his therapy goal. This case study provides support for a psychological intervention to support the psychological rehabilitation after an ABI.

## Introduction

A moderate-to-severe acquired brain injury (ABI) can have tremendous lifelong consequences for ABI-survivors and their families. Many ABI-survivors are facing challenges of physical disability, cognitive deficits, and psychosocial sequelae. It has been suggested that especially psychosocial problems can be a major challenge for people with ABI, both during and post the rehabilitation process. Some of the most common psychosocial consequences are anxiety and depression, which impacts between 19.5 and 79% of the population (1, 2), and identity reconstruction or “loss of self” (3–7). Despite rehabilitation practice since the 1980s aspiring to a dynamic, coherent and holistic approach, addressing the life changing transitions caused by an ABI, the psychological dimension still seems to be a challenge and research has revealed persisting psychosocial impairments after ABI (5). Rehabilitation originates from a biomedical field (the medical model) where identification of pathology was seen as a first step to problem solving (8). Rehabilitation practices and research are still mainly influenced by rehabilitation's origin in physical medicine. However, modern medicine has a blind spot. With its dominant focus on pathology, it ignores the existential dimension of life-changing illness and its impact on emotional wellbeing. This is unfortunate since rehabilitation is more than the rehabilitation of the body and the brain. A rehabilitation program should also enable the individual to live his or her life, in a way so it becomes possible to participate and contribute to family life, community and society (9).

Because of the complexity of physical, cognitive, and emotional impairments following an ABI, rehabilitation efforts must be comprehensive and multidisciplinary including nursing care, physiotherapy, speech and language therapy, clinical psychology or counseling, vocational and social support (10, 11). The purpose of a multidisciplinary approach to rehabilitation after ABI is defined by the ABI-survivors' symptoms and can therefore vary, but often it incorporates close cooperation, awareness of communication and sharing of knowledge within the team, in regard to the professionals involved and a two-way interactive process between ABI-survivors, their families and an interdisciplinary team of professionals (12, 13). Even though holistic rehabilitation is an international recommendation (14), no clear agreement about the content of rehabilitation interventions or programs is evident from the literature. Furthermore, the rehabilitation settings, program goals, and outcome measures vary in character and content. As well as the lack of detailed descriptions of psychotherapeutic interventions and the role of psychotherapy in brain injury rehabilitation is unclear (15–18). Despite the heterogeneity of interventions, a few psychological therapies are on the rise in the field of neuropsychological rehabilitation that offers positive treatment results on a variety of cognitive, emotional, and interpersonal changes after ABI.

To date, the most established psychological intervention that offers relief of some of the psychological sequelae symptoms after ABI is Cognitive Behavioral Therapy (CBT). A systematic review by Cicerone et al. (19) found that CBT was used to increase coping behaviors and reduce emotional distress, improve executive aspects of attention and self-reported everyday functioning. Similar findings were described in a more recent systematic review by Gallagher et al. (20) on cognitive impairments following brain injury. Gallagher and colleagues identified different types of CBT that were modified to treat post-injury cognitive and emotional problems. Their findings suggest that psychoeducation based on the CBT model is one of the most frequently used adaptations because it gives ABI-survivors the knowledge of their own changed cognitive capacities and helps to create new patterns of thinking that eventually can influence behavior. Moreover, written formulations and homework tasks, as well as coping thoughts and relaxation exercises were also commonly used. This was done to accommodate problems with attention, enhance homework compliance and reduce anxiety. The 18-item Modification-Extraction List displays the most commonly reported adaptations of CBT (20). Indeed, some of the adaptations describe the core of the CBT-model, which is based on the premise, that information received from our own bodies and the outside world can influence the experience of emotions, thought processes and behavior (21).

Within ABI therapeutic interventions, there has been a development in recent years of using “third wave” cognitive behavior therapies, which emphasize self-compassion, compassion for others and the ability to be sensitive to the compassion from others through attention-awareness and motivation. Compassion Focused Therapy (CFT) (22) focuses on self-accept using appropriate or desired value-based life strategies by means of engagement in actions that lead in those directions. Moreover, Acceptance and Commitment Therapy (ACT) (23) seeks to develop greater awareness of thoughts and feelings as mental events rather than as reflections of reality through meta-cognition and meditation. However, a review of the literature reveals a small body of evidence of “third wave therapies” effect on psychosocial sequelae after ABI so far (24). The studies to date using “third wave therapies” interventions after ABI have demonstrated an improved quality of life (25), reduced symptoms of anxiety and depression (26–28), fatigue (29) and significant reduction in measures of self-criticism (27).

The research outlined above indicates that “third wave therapies” has potential as intervention for ABI-survivors, but there is still a sparce body of research. Thus, there seems to be a need for more research in the area. Furthermore, to our knowledge, no previous studies have examined the effect of combined use of the aforementioned “third wave therapies” that potentially may give more flexibility in therapeutic interventions with the purpose of meeting the challenges, which adults with ABI may experience during rehabilitation.

Therefore, the present pilot study provides the first attempt to explore the effect of a manual-based intervention program (BackUp) developed specifically to support adults with ABI through the psychological reactions that may follow an acquired brain injury. Furthermore, the study seeks to explore a client's experience of psychological rehabilitation during the rehabilitation process after ABI.

## Methods

### Design

For the purpose of the study, a single-case A-B-A design was implemented. The design involved the assessment of several measures at a baseline before the initiation of the intervention phase and then the assessment was repeated after the intervention and at 9 months follow up. Throughout intervention, consisting of 12 sessions, a measure of “daily rehabilitation progress” was implemented as an evaluation tool to assess participants' daily progress.

### Procedures

Since the study involved human participants and person-sensitive data, the study was approved by the Danish Data Protection Agency (Datatilsynet). The project was also reported to the Regional Research Ethics Committees for the Region of Northern Jutland (Nordjylland) who found it exempt from full review. Informed written consent from the participant was obtained at the screening phase. The pre, post, and follow-up assessment measures were administered by the leading therapist of the psychological intervention with the help of the professional staff at the neurorehabilitation center due to the participant's cognitive challenges.

The BackUp intervention was delivered by a trained clinical psychologist with several years of experience with ABI-survivors. Each session was observed and recorded by a psychology master student from Aalborg University, to monitor treatment fidelity of the treatment (30). The intervention period was 3 months, sessions were given once or twice a week depending on participants' program agreement. Sessions were held in a therapy room at the rehabilitation center. The therapy room was used by this psychologist only.

### Measures and Analysis

The four standardized self-report measures were administered during the first and last sessions of the intervention program with the purpose to provide evidence for treatment effectiveness. In a classical A-B-A design assessment is also necessary during phase B. However, answering the primary outcome measures are comprehensive and can be difficult for people with acquired brain injury, therefore we developed a secondary outcome measure, for this specific intervention program, to be used on a daily basis during phase B. The secondary outcome measure was a daily self-report questionnaire measuring rehabilitation progress. A similar design was used by Hsieh et al. (31). The purpose of the secondary outcome measure was to look for possible patterns that might suggest specific links between the intervention parts and the rehabilitation progress.

Nine moths post intervention a follow-up assessment was conducted using the same primary outcome measures and a semistructured interview exploring the participants experience of the intervention. For the analysis of the data, spaghetti plot was used for visual inspection of the data (32).

#### Primary Outcome Measures

##### Depression and Anxiety

The Danish translation of the Hospital Anxiety and Depression Scale (HADS) is a brief self-report measure (14-item) that contains seven intermixed items for both anxiety and depression. The questionnaire was originally developed by Zigmond and Snaith in 1983 to shift attention from the physiological symptoms to behavioral components of anxiety and depression and have previously been used to evaluate symptoms of depression and anxiety among people with acquired brain injury [e.g., (27, 33)]. Symptomology of anxiety and depression are rated on a scale of 0–3, on which the highest score indicates the severity. The total score is calculated for each scale and ranges from 0 to 21. For either subscale, a score from 0 to 7 is regarded as normal, 8–10 mild, 11–14 moderate and 15–21 severe (34).

##### Personal Growth

A Danish translation of the Personal Growth Initiative Scale (PGIS) was administered to capture change and growth in the rehabilitation process that could be facilitated by the challenges from the ABI trauma, new ways of coping with cognitive and physical disabilities as well as the psychological intervention. The PGIS is a nine-item self-report measure scored on a 6-point Likert scale (1 = “strongly disagree” to 6 = “strongly agree”). Possible scores range from 9 to 54, with higher scores indicating greater growth initiative. Items assess the three possible ways of growing: unintentional and out of awareness, unintentional but in awareness and intentional and fully in awareness. The measure has robust psychometric properties (*a* = 0.87) (35, 36).

##### Quality of Life

The 5-item World Health Organization Well-Being Index (WHO-5) contains five positively phrased statements about respondents' subjective wellbeing for the last 14 days. Each item is scored on the 6-point Likert scale, from 0 (“none of the time”) to 5 (“all of the time”) with the raw scores ranging from 0 to 25. To calculate the total score, the raw score is recommended to be multiplied by four, which represents a percentage scale ranging from 0, indicating the worst imaginable wellbeing to 100, indicating the best imaginable wellbeing. WHO-5 was first published in 1998 and since then has been translated into more than 30 languages, including the Danish language. The questionnaire has been used both as a screening tool for depression and as an outcome measure in clinical trials and has a strong sensitivity of 0.83 and specificity of 0.81 (37).

##### Compassion for Oneself

For the primary outcome measures, the Self-Compassion Scale (SCS) consisting of 26 items were used in the pre-test and the follow-up. It is based on the three basic components of self-compassion, which entails (1) kindness and understanding toward oneself; (2) seeing life experiences as a journey to better understand one-self, and (3) an ability to avoid over-identification with negative thoughts and emotions. SCS comprises the three positive subscales: *Self-Kindness Subscale, Common Humanity Subscale, Mindfulness Subscale*, and three negative subscales: *Self-Judgment Subscale, Isolation Subscale*, and *Over-Identification Subscale*. Each subscale score calculates separately with a reverse coding response to the negative subscales. An overall self-compassion score is then achieved by summing the means and dividing it by 6. The original SCS demonstrates a good factor structure as well as construct validity (38).

For the post intervention outcome measures, the Self-Compassion Scale-Short Form (SCS-SF) was implemented instead, to accommodate cognitive limitations caused by the ABI. SCS-SF is a 12-item measure of self-compassion. Despite its reduced length, it is still a reliable scale with the same factorial structure as the long version of SCS. Correlations between the corresponding subscales for the original and short versions of the SCS range from *r* = 0.84 to *r* = 0.93, the total score correlation is *r* = 0.97 (39). Both scales were translated by researchers from the Center for Development and Applied Psychological Science at Aalborg University in Denmark.

#### Secondary Outcome Measure

The self-report questionnaire measuring daily rehabilitation progress was developed for this study to evaluate and find patterns in the holistic rehabilitation program. The questionnaire consists of nine questions with responses given on a horizontal VAS scale from 0 to 10, where 10 is the best imaginable. A similar technique was used in a previous study by Rasquin et al. (40). The questions are presented in Table 1.

**Table 1:** Questions for measuring daily rehabilitation progress.

**Number**	**Questions for measuring daily rehabilitation progress:**
Q1	How is your mood today?
Q2	How self-critical have you been today?
Q3	How satisfied are you with yourself today?
Q4	How well has your body functioned today?
Q5	How satisfied have you been for your life today?
Q6	How well did you sleep last night?
Q7	Did you want to be social with others today?
Q8	Have you taken the initiative to be with others today?
Q9	How well has your memory functioned today?

### Interview

In relation to the follow up assessments, a semi structured interview was conducted by a research assistant, who had not previously been a part of the project. The interview sought to clarify the participant's experience of the psychological intervention at the rehabilitation center. The primary interest was the experience of the individual intervention based on the BackUp program. However, it was also of interest to explore the participant's experience of his psychological rehabilitation at the rehabilitation center in general. After ending the individual intervention program following the BackUp manual, the participant received additional sessions with the therapeut and participated in a group intervention facilitated by the same psychologist and a musictherpeut. It was expected that these following interventions would also affect his experience. However, in this study, the following group intervention are understood as part of the holistic rehabilitation program.

The interview was conducted at a vocational rehabilitation center which offer users a voluntary association. The research assistant worked there, and the participant was a member.

### Follow-Up Assessment

In agreement with the participant, self-report measures were sent to him prior to the interview. However, he experienced, that he needed help to fill out the tests. Due to restrictions following the COVID-19 situation, it was agreed between the first author and the participant, that the first author was to call the participant and ask the question over the phone, after the interview. A similar procedure has been used in other studies [i.e., (41)].

### Participant

The participant was a 58-year-old man attending a neurorehabilitation program at a specialized inpatient rehabilitation center for people with moderate to severe brain injury. We refer to him as Lars instead of his real name. He was offered a manualized psychological intervention as a part of a holistic rehabilitation program after his ABI. Lars had suffered multiple infarcts in the right hemisphere. After having been hospitalized at two different hospitals, he arrived at the rehabilitation center 6 months post injury. At that time, Lars was in a wheelchair, but during the psychological intervention he gained the ability to stand and take small steps with support. Besides his mobility challenges, he suffered from neglect and profound executive difficulties including challenges with overview and structure. In addition, visuo-perceptual and spatial difficulties were seen. At the rehabilitation center, Lars was assigned to a team of physiotherapists, occupational therapists, educators, caregivers and a neurologist, all with special knowledge of neurorehabilitation. Rehabilitation was planned and incorporated in all parts of his daily life at the rehabilitation center. Lars's team experienced Lars as having lack of insight in his ABI which caused many disagreements between the staff and Lars. The psychologist's assessment of Lars was that what seemed to be lack of insight, was also due to a defense mechanism (42). In cooperation with the municipality, the rehabilitation team and Lars, a main goal for his rehabilitation stay was formulated; That Lars becomes as independent and safe in his home as possible, so that he can function alone in his own home. For Lars the most important goal is, that he wants to be able to go out fishing again. Preinjury, Lars had an active outdoor life and enjoyed fishing multiple times a week. Moreover, he is a very social man, with a physically active work in the service industry for 40 years.

Due to the disagreements, in relation to what was perceived by the profession team as Lars' lack self-awareness, the psychologist was included in the rehabilitation process. After initiating supervision of the team, it was hyphotized that Lars would match a manual-based intervention program based on the BackUp program.

## Intervention

### The BackUp Intervention Program

As discussed in the introduction section, ABI-survivors frequently face emotional consequences such as anxiety and depression as well as agentic and identity issues. The aim of our BackUp intervention program is to address these issues.

Two of the authors developed the first versions for use by psychology students during their internship in clinical practice in the field of ABI (43). Based on clinical experiences, minor adjustments were continuously made, until the first author finalized the current version presented here.

Within an overall framework of 3rd wave Cognitive Behavioral Therapy [cf. (44, 45)], the program covers themes that often need to be addressed for ABI-survivors, and it comprises selected CBT and narrative intervention techniques that can be used with this client group. The rationale for these choices was alluded to in the introduction section of this article and is further elaborated in the section below. Table 2 provides an overview of themes and techniques by session.

**Table 2:** Overview of interventions in the BackUp program, by session.

	**Session title**	**Session goals and exercises**	**Mindfulness exercises**	**Homework activity**
1	Pre-interview with assessment and initial case formulation	Framework Psychoeducation: brain injury, rehabilitation, transition Assessment: WHO-5, SCS, HADS, PGIS	Short body scan	
2	Psychoeducation about brain injury	Psychoeducation: biopsychosocial model of health, major rehabilitation models, cognitive sequela after ABI, (mental) fatigue		Weekly schedule planning and registration, “goal stairs”
3	Psychoeducation: emotional reactions	Psychoeducation: crises responses and grief reactions Education about identity change Depression and quality of life		Registration of activities that give or take energy
4	CBT	Case formulation and defining important issues Psychoeducation: CBT-model, education about automatic negative thoughts	Short body scan	Daily activities schedule, registration of negative thoughts
5	CBT	Continuation from the previous session Work with client-chosen topics or alternatively with suggestions from the therapist	3. min. breathing exercise	Meaning: What makes life worth living anyway (ACT worth chart)
6	Introduction to Compassion-Focused Therapy (CFT)	Education and work with Gilbert's model Topic: “You have a tricky brain and it is not your fault” Goal: treat yourself properly when you face difficulties - no matter whose fault it was	Compassionate breathing exercise	Self-compassion journal
7	CFT	Continuation from the previous session Exercise: Compassionate letter to yourself Topics, e.g., It is not fair, It was my fault Goal: Learning to let it go—inner workings with self-criticism	Compassionate breathing exercise	Self-compassion journal
8	CFT	Continuation from the previous session Exercise: Visualization with a compassionate figure	Compassionate breathing exercise	Self-compassion journal
9	CFT	Continuation from the previous session Exercise: Compassionate reminiscence Topic: Family—guilt/shame/self-criticism	Compassionate breathing exercise	“Important and most important”—What was important in your life at a different age?
10	Identity reconstruction and quality of life	Topics: Before and after ABI trauma—the quality of life, identity, values, and meaning of life Exercise: All behavior is assessed through the magnifying glass		
11	Identity reconstruction	Continuation from the previous session Exercise: 90th birthday Goal: Get in touch with personal values	Short body scan	
12	Feedback	Assessment for post-tests and feedback		

#### Design Rationale

Cognitive behavior therapy (CBT) is the most empirically validated form of psychotherapy. Findings derived from randomized control trials provide support for the empirical validity of CBT demonstrating that CBT is as effective for the treatment of depression as pharmacotherapy [e.g., (46)]. CBT has been shown to be efficacious for the treatment of a variety of psychological consequences but despite the vast literature demonstrating this, relatively few studies have focused on the development of a specific CBT intervention for improving mood and coping after brain injury (47–49). Nevertheless, in addition to being the empirically validated treatment of choice for a range of psychiatric disorders, there is an increasing amount of literature illustrating that CBT can be successfully adapted and applied to a diverse set of neurological and medical populations (50). Anson and Ponsford (51) evaluated the effectiveness of a CBT-based coping skills intervention for 31 individuals with TBI. The intervention was designed to improve both coping strategy selection and emotional adjustment. Following treatment, participants demonstrated a significant improvement in adaptive coping.

To complicate matters, previous research has found that issues of self-criticism and shame are frequent after ABI (52) and may interfere with the treatment. In a study from 2011, Fiona Ashworth and colleagues treated a client with symptoms of depression and anxiety with classic CBT but found the intervention to be ineffective due to the client's self-criticism. They therefore changed the approach to Compassion Focused Therapy, which led to an effective reduction in symptoms for the client (53). Mindfulness, acceptance, and self-compassion are core issues in 3rd generation CBT, and the BackUp manual suggests a number of techniques to help the client achieve this, most of them from Compassion Focused Therapy.

Moreover, recent research also emphasize that rehabilitation psychology needs to address identity reconstruction as well as emotional adjustment after ABI. A growing body of research has shown a potentially vast impact of an ABI on identity and agency that may hamper psychological and social recovery [e.g., (7, 9)]. Narrative techniques were included in the BackUp program to address identity issues.

To summarize, the themes included in the BackUp program are based on previous research and clinical experiences, and to address them, techniques from 3rd generation CBT are supplemented with narrative techniques.

A recent review by Salas and Prigatano (54) argues for a integrative approach to therapy after acquired brain injury, to accommodate the specific needs of the clients (54). The BackUp program was developed before that review but seems to match its recommendations.

#### BackUp Structure and Use

The BackUp program involves 12 individual therapy sessions, which are delivered once or twice a week with ~45 min each. The intervention program is manualized, and the content includes several aspects from 3rd wave cognitive therapies, such as psychoeducation, mindfulness, and compassion exercises, as well as addressing existential and identity issues with narrative techniques (Table 2).

Most sessions have the following structure: welcome, follow up from the last session, new topic, exercise, and homework. Considering previous research and participant's particular rehabilitation goals, different strategies are implemented to accommodate cognitive impairments; for example, changes in sitting position, different communication tactics, and presenting psychoeducation in multiple formats, such as drawing on the paper and providing relevant handouts.

Due to the heterogeny of ABI clients, the therapist is encouraged to sample from the techniques and themes of the manual as clinically relevant for the individual client's challenges and resources.

### Case-Adapted Implementation of the Manual

The aim was to implement the manual so that all themes in each session is addressed during therapy. At the same time, the manual has been developed so that it must be adapted to the individual client's needs and wishes, as well as the agreed therapy goals. Overall, the intervention was given in accordance with the manual, with small alterations to the client's individual needs. For example, more time was spent on psychoeducation related to themes relevant to the client, while the client was encouraged to read about the other topics in the handouts as homework. Another change was made to the homework. The client did not do the homework exercises in the beginning of the intervention. He found registration and journaling difficult, and he was often dissatisfied with his own performance and ended up throwing out his notes before therapy. Between sessions he reported having many reflections about the exercises and he completed the questionnaires daily, so it was agreed that it was more important that he reflected upon the exercises than writing them down.

## Results

This study aimed to investigate a psychological intervention program for a person with ABI as a part of a holistic rehabilitation program at a specialized rehabilitation center. Changes was measured at three time points (pre-intervention, post-intervention and at 9-month follow-up) using the self-assessment four scales (HADS, WHO-5, PGIS, SCS). Assessment scores can be seen in Table 3.

**Table 3:** Pre-, post- and follow-up scores on assessment tests.

**Assessment**	**Pre intervention**	**Post intervention**	**Follow-up (9 months post intervention)**
HADS depression	6	10	4
HADS angst	0	4	3
PGIS	46	54	54
WHO 5	68	24	72
SCS total	17,2	17,5	17,4

**Figure 1:**
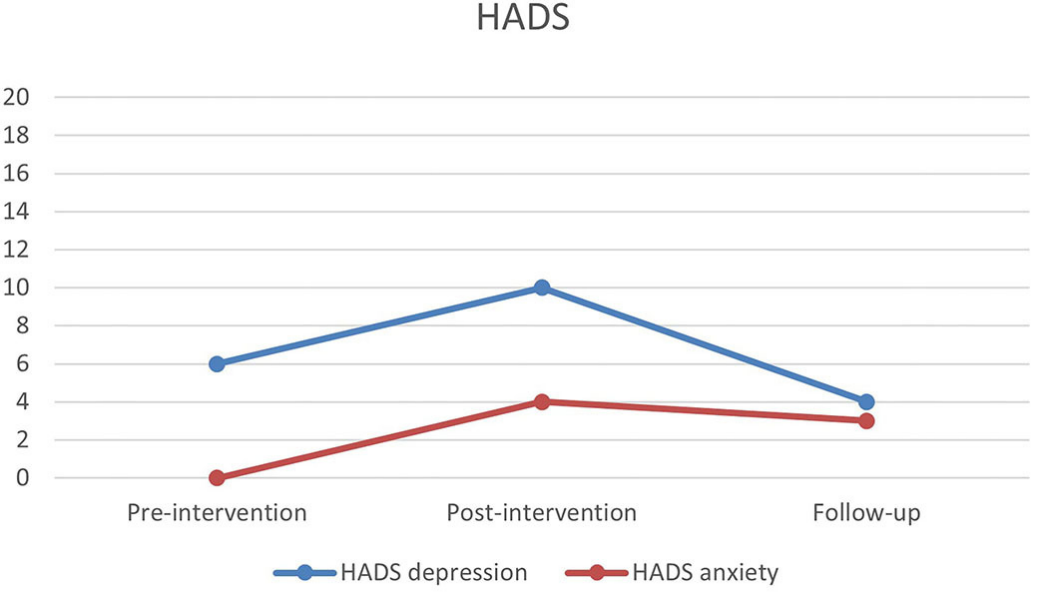
Pre-, post- and follow-up scores on Hospital Anxiety and Depression Scale (HADS).

Figure 1 shows changes in depression and anxiety symptoms during all three assessment points. Depression scores increased from the pre-intervention to post-intervention, from a raw score at 6–10, which corresponds to non-depression to mild depression according to applicable cut-offs. At 9 months follow-up, the raw score was 4, non-depression. Scores at the anxiety scale was at all three time points below cut-off for signs of anxiety.

**Figure 2:**
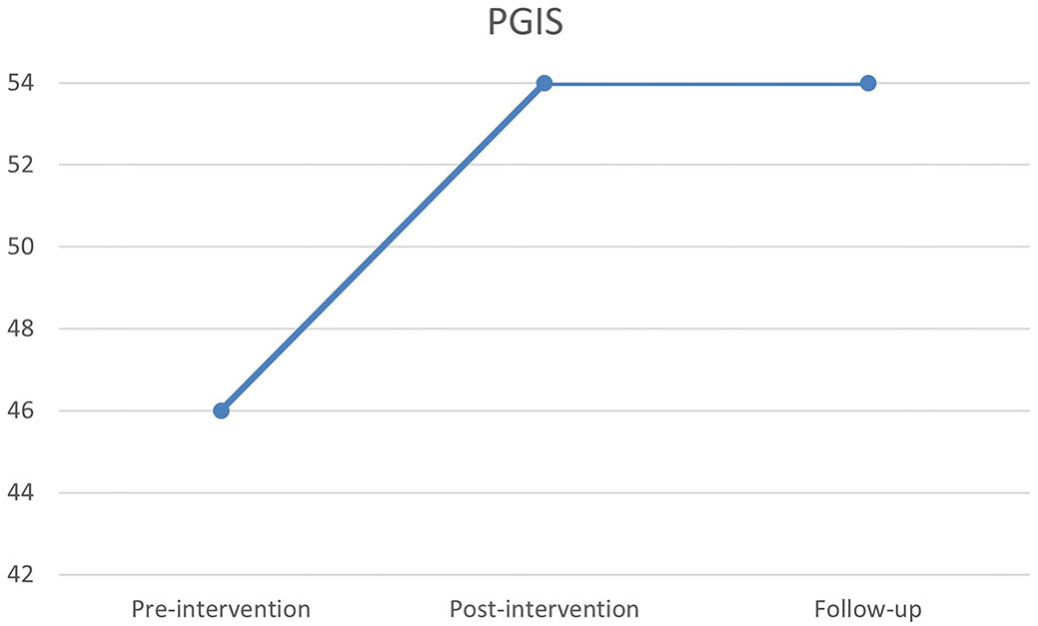
Pre-, post- and follow-up scores on Personal Growth Initiative Scale (PGIS).

An indicator of Lars' agency was obtained by the Personal Growth Initiative Scale (PGIS). Figure 2 shows an increase from pre-intervention to post-intervention and follow-up, going from a raw score at 46 to the maximum of the scale at 56, being stable between post-intervention and follow-up measurements.

**Figure 3:**
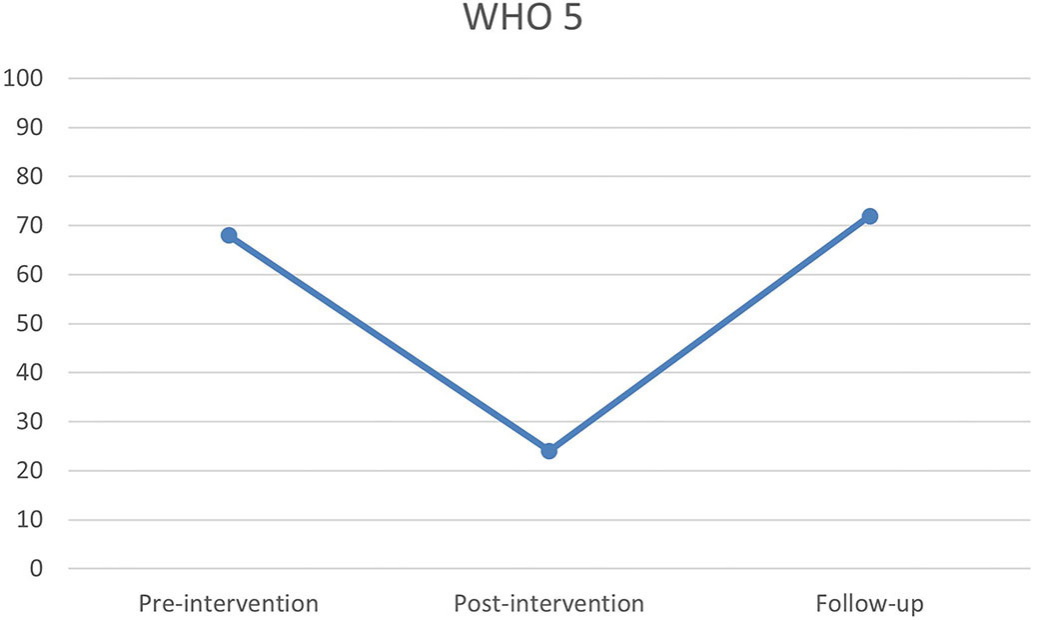
Pre-, post- and follow-up scores on the 5-item World Health Organization Well-Being Index (WHO-5).

Quality of life was measured using the 5-item World Health Organization Well-Being Index (WHO-5). During the three time points of measurement raw scores interpreted by cut-offs moved from normal at pre-intervention, to lower than average and a need for awareness at post-intervention, and at follow-up scores had returned to normal (Figure 3).

The Self-Compassion Scale (SCS) was used to evaluate the changes in relation to self-criticism. Raw scores are seen to be stable over all three time points.

### Evaluation During Intervention Period

**Figure 4:**
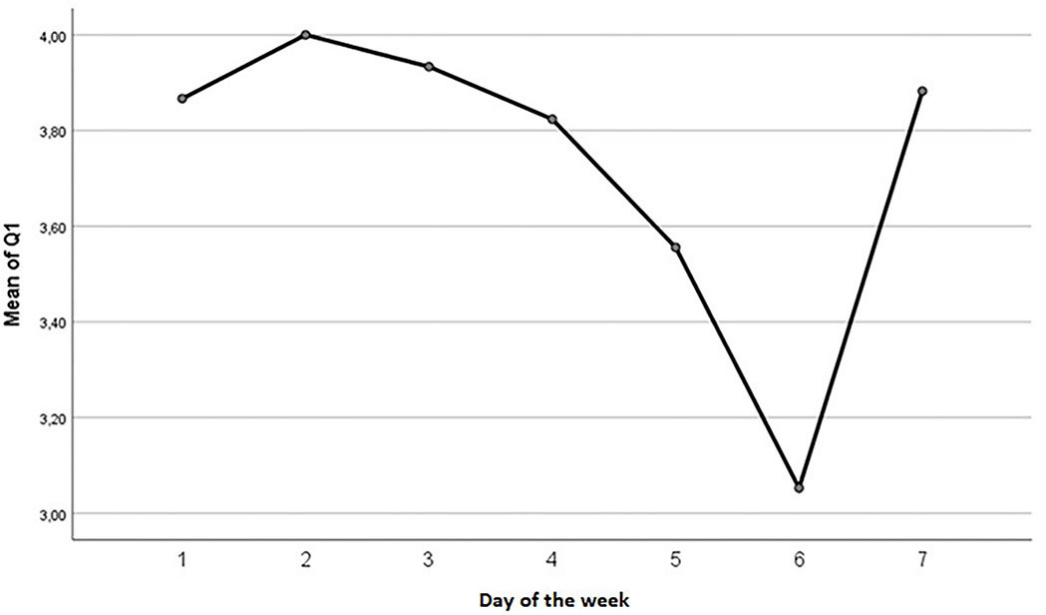
Plotted mean scores for question one, over the course of the week, where 1 represent Monday, 2 Tuesday, etc.

A one-way ANOVA revealed no significant differences between the different days and the different questions. However, an interesting trend seem to show in a visual inspection (Figure 4). Therapy sessions were always held on Mondays.

Six out of eight questions have lowest mean scores on Saturdays, while the remaining two questions score lowest on Mondays or Tuesdays (Table 4). This suggests that up to and immediately after the day of therapy (Monday), the client provides highest responses, while the weekend day Saturday receives the lowest scores. No significant change was found between the difference in the highest and lowest value. For example, a one-way ANOVA showed that F = 1.198, df = 33, *p* = 0.282.

**Table 4:** Highest and lowest mean scores for the daily questions, by weekday.

**Question**	**Day with lowest mean score**	**Mean/SD**	**Day with highest mean score**	**Mean/SD**
1	Saturday	M = 3.1; SD = 2.3	Tuesday	M = 4; SD = 2.7
2	Monday	M = 3.5; SD = 2.2	Thursday	M = 4.1; SD = 2.7
3	Saturday	M = 1.7; SD = 2.1	Tuesday	M = 2.9; SD = 3.4
4	Thursday	M = 1.0; SD = 1.0	Sunday	M = 1.6; SD = 1.9
5	Saturday	M = 2.1; SD = 2.3	Sunday	M = 3.5; SD = 3.9
6	Saturday	M = 6.7; SD = 2.3	Monday	M = 7.7; SD = 1.7
7	Saturday	M = 3.7; SD = 2.6	Monday	M = 4.4; SD = 2.0
8	Saturday	M = 2.3; SD = 2.4	Wednesday	M = 3.1; SD = 2.4

### Therapist's Evaluation

The therapy goal, for Lars, was to recognize his own limitations in order to be able to participate in the rehabilitation program and to prevent conflicts with the rehabilitation team. During the last session of the intervention program, the psychologist and Lars qualitatively evaluated the intervention regarding the goal of supporting a process of self-awareness for Lars. Lars expresses that he has gained more insight into the changes of his life following the brain injury. During the therapy Lars was able to reflect on his new life circumstances and work with his values and identity reconstruction. Lars and the caregiving team no longer experienced the same degree of disagreements in the collaboration and Lars had become more independent. Lars was seen to gain more from the physical training when he had control over his own situation. It was assessed that Lars could benefit from an extension of his stay at the rehabilitation center, which he accepted.

### Evaluating Interview

The interview was analyzed using Thematic Analysis (55). During the analyses of the follow up evaluating interview, the following themes appeared: *Fellowship, security, individual therapy, group therapy, Quality of Life, holistic rehabilitation, training, the rehabilitation center, inside perspective*.

#### Fellowship and Security

During the interview, two central themes emerged: the importance of fellowship and security. It is clear how fellowship and social interaction was an important thing for Lars, not only during the rehabilitation process, but also a central theme in his life. For Lars, fellowship had many functions. For example, fellowship with other clients at the rehabilitation center was a source of motivation:

“*I: What did it mean you had someone to go with? L: It meant that the motivation to go was greater.”*

Therefore, it was important for him to have group activities with peers at the center. During the interview, he highlighted a wish for more group activities at the rehabilitation center. In a similar vein, Lars highlighted the group therapy that he attended at the center after the individual psychological intervention. The opportunity to learn from peers and share experiences was one of the benefits he highlighted:

“*I: Yes: So the thing about passing on the experience L: Yes I: It's something you've experienced, yes: L: That's also why it's so good – those conversations you have, to sit and talk”*

The fellowship is not just about how to share and learn from others in the same situation, it is also about caring for others. This relates to the second theme: *security*.

“*L: Security, it's incredibly important”*

Lars mentions how security is important in various settings, that is in the environment, in the training, in relationships and in groups. In general, security is important for Lars and has a huge impact on the benefits he gains from different activities and relationships during his rehabilitation. Regarding the relationship with the psychologist and the music therapist Lars expresses:

“*L: […] I was very comfortable with them. I: Yes L: It also matters I: So you've got – you've got some good experience – L: Yes, I have.”*

#### Therapy, Inside Perspective and Holistic Rehabilitation

Lars does not mention the outcome of the individual therapy on his own. However, when the interviewer asks Lars about the therapy, he says he has been very happy with the intervention and that he feels that it has had a big impact on him.

As mentioned in the presentation of Lars, he often experienced disagreements and conflicts with the caregivers, in relation to self-awareness or insight into the consequences of the brain injury. In the interview, Lars express how he was challenged when his deficits were in focus, rather than his potentials. According to Lars, he was aware of his challenges, but a focus on challenges was not supportive for him.

“*L: And then there was one – I think it was the second time I was at that meeting, So – I got up and walked, I didn't, I: Uh: turned around and backed out, and then I said - what's it called – ”I think you should, at these meetings, talk about what you can do for me, I know what I can't do, you don't have tell me that, I know this very well”*

The therapeutic goal of the intervention was working with insight and acceptance, this is also one of the outcomes Lars points out when asked:

“*I: So what you're talking about now, that's some insight, and some acceptance of your situation L: Yes, my own situation, yes I: Yes. Has it had an impact that you have talked to [the psychologist] and the group? L: Yes, that it has I: Yes L: That you also come: - if one: is – accepting to oneself, that you come the furthest by being honest and then realize the things that are.”*

Lars concludes that he experienced the psychologist, as well as other key professionals around him, as being very caring, and that it was very important to him. In relation to the individual therapy and the subsequent group therapy, he states that the two interventions were important:

“*I: Yes, two interventions L: Yes: I was very* ***happy with them*.**”

He elaborates about the individual therapy:

“*I: […] And what about [the psychologist]? L: I think she went more into depth, personally. I: Yes. L: Or I think - she* ***did****.”*“*L: We talked about everything. It was so nice and easy. (.) She's easy to talk to. I: Yes. How is one too easy to talk to? L: Just like we do now. I: Yes. What happens when you: uh: when you're easy to talk to. What is it? L: I think the most important thing is feeling safe. I: Yes.”*

During the evaluating interview it became apparent that the intervention was feasible, and that Lars was satisfied with the intervention.

For Lars, the experience of receiving psychological rehabilitation is well-related to the physical training.

“*L: Because, as we talked about before with the psychological and the physical, it's related. I: Yes. L: It helps each other I: Yes L: If one does not work, the other does not work either I: No L: Therefore, I believe that it: - there is too much focus on physical training. You'd have to think more about the psychological part”*

It is clear from the interview that a balance in the bio-psycho-social model is important to him. Fellowship and relationships are important to Lars as well as the need for physical training. It is seen that both biological, social and psychological factors have an impact on overall rehabilitation.

“*L: But if the mental part is not okay, then the physical cannot be either.”* “*L: So it's a combination. There's no point in being trained to walk, if you are not trained in: that: uh, the head - it must work as well.”*

In addition to the individual therapeutic intervention, Lars participated in a group therapy during his stay at the rehabilitation center. The group therapy was facilitated by the rehabilitation center's psychologist and a music therapist, drawing on intervention methods from both approaches. The group therapy was something Lars returned to several times during the interview. It is something that has made a great impression on him, especially in relation to the social fellowship of the group. The group represented security for him, and it was important that they had something in common.

#### Training and Rehabilitation Center

During the interview Lars talked about other experiences he had during his stay at the rehabilitation center. Experiences in everyday life was important for the training and recognitions of changes following the brain injury.

“*I: There was a day when I had to make soup. I had prepared it in the morning. So, for dinner, I chose to turn it on, and then I went over to train in the gym. Then came [name] over, one of the educators said “L, I've turned off your soup, I think it's finished” I: Well yes L: And then I came to think of, “L now you better, when you come home, you better get someone to come in and look, if: you've started something” I: Yes: L: For there I said to myself, it may be one of your injuries:that:that you've started something” I: Yes: L: For there I said to myself, it may be one of your injuries: that: that you do it. I: Yes. Yes, other people also experience that. That they go from something, they simply forget that they've turned it on. L: Well my problem: and what annoys me, it is: is that I knew it well, because I turn it on, and then I go I: Hmm”*

Having concrete examples of everyday challenges can help to detect them and later see how you progress in overcoming them. It opens a subsequent opportunity to work on the challenge and actively train it in everyday life and learn new strategies:

“*L: But I have also had my [family member] to make some notes and put up “Remember to turn off” signs I: Yes. So there are some strategies you've got, to: and: help you remember to switch off L: Yes, yes, exactly. They hang so I keep watching them I: Yes.”*

Not only planed exercises during the rehabilitation, but also concrete everyday experiences, have helped Lars recognize his own difficulties and take the necessary precautions.

## Discussion

This case study set out to explore the effect of a psychological intervention program, as well as how a client experiences the intervention after an ABI. While results from pre, post and follow-up measures does not show clear positive results, the interview reveals positive experiences with the intervention. Previous studies in the area show similar unclear results measured by quantitative parameters, while clients express good experiences of interventions [e.g. (56, 57)]. No significant fall in symptoms on the HADS assessment could be expected, since Lars did not complain about depressive or anxiety symptoms before the intervention and the assessment did not show signs of either pre intervention. However, results of the two other assessment tests, which indicated no changes for SCS and a decrease on WHO-5, is more surprising. It should be considered if the measuring tools were inadequate, or the client simply did not have quantifiable benefits—including whether the specific client is a special case. Furthermore, it can be argued that an effect cannot be expected on a client who does not experience greater symptoms of psychological distress. Why even intervene if the client does not rapport symptoms of either depression or anxiety beforehand. However, as argued in the introduction the rationale for a psychological intervention should not only be found in the prevalence of depression and anxiety among people with acquired brain injury, but also in the existential changes during a crisis. A psychological intervention is expected to not only lower symptoms of depression and anxiety, but also increase the overall outcome of the rehabilitation.

Based on this consideration, it can be argued that the intervention should be evaluated in relation to the therapy goal to a greater extent than based on symptom reduction. A previous study by Gracey et al. (57) highlighted the importance of evaluating future studies of the effect of psychotherapy for this group of clients on the bases of achieving the therapy goal. Furthermore, the third wave cognitive therapies would argue, that the goal of the therapy should not be symptom reduction but recreating a sense of meaningfulness in life (58).

The qualitative therapy goal, for Lars, was to recognize own limitations in order to be able to participate in the rehabilitation program and to prevent conflicts with the rehabilitation team. This goal was achieved as noted by both Lars and the psychologist. Furthermore, Lars was able to better collaborate with the rehabilitation team and was offered an extension of his stay at the rehabilitation center for further 3 months after the intervention, which he accepted. This allowed him to further improve in physical and cognitive functions. In this light, the intervention was effective, and it can also explain the results in personal growth (PGIS), on which Lars showed a great increase in agency. On the other hand, results from the HADS assessment showed increased symptoms of depression and anxiety post-intervention, and the results from WHO-5 showed reduction in quality of life. This apparent discrepancy may be explained by a relapse, which is to be expected when becoming aware of one's own limitations. This could further cause a grief reaction (58). Perhaps the increase of depression and anxiety symptoms can be understood as a reaction to his increased insight into own limitations, and as signs of a grief reaction. Very little is known about how people with ABI experience grief, and it has been suggested that symptoms of depression can be understood as such (59). At the time of the follow-up all scores had stabilized at a non-pathological level. From previous studies, it is known that the insights obtained during therapy for some people with ABI takes longer to consolidate (40). During the interview Lars stressed the importance of the therapeutic relationship, and a study by Zelencich et al. (60) argues for a link between a good therapeutic relationship and increased self-awareness.

If increased insight or self-awareness increases the risk of anxiety and depression, one may question why a psychological intervention should seek to support an insight process, since depression and anxiety post brain injury are associated with poorer functional outcomes (61). As reported above, lack of insight can hinder the rehabilitation process in general (58). Therefore, it can be suggested that symptoms of depression or anxiety in a period of the rehabilitation process can occur as part of a natural grief reaction (59, 62). Since reactions of grief is commonly seen in relation to increased self-awareness and identity reconstruction following the brain injury (58, 63).

During the intervention period, the participant completed the self-report questionnaire measuring daily rehabilitation progress. As seen above no significant results was found from this questionnaire. This may be due to different problems. First, *N* = 1 which means the data set probably is too small, and the scale has not been calibrated. Another problem might be in the definition of the week beginning on a Monday. From the visual inspection of question 1 it appears that had we started the week on a Sunday, a regression might have appeared.

The objective of the secondary outcome measure was to identify patterns that could link specific intervention aspects to rehabilitation progress. This was not possible, and it can be argued that another measurement would be more suited.

### Future Directions for Interventions and Society

Based on the presented case, we recommend that psychological interventions should be given as an add on to existing holistic rehabilitation programs to enhance the overall rehabilitation outcome and support the client's psychological adjustment to changes after an ABI (59). Moreover, society should acknowledge the importance of psychological support following ABI, in order to support the total outcome of holistic rehabilitation. As Lars expresses the psychological rehabilitation and the physical rehabilitation are inter-dependent.

An ABI is a life changing event. An individual not only loses bodily functions, but also social and relational positions (work, network, family, etc.). The recognition of our shared humanity is crucial. Instead of dehumanizing and pathologizing ABI survivors, normalization could be an important aspect to consider further, which implies a basic mutuality in the experience of suffering. As long as there is so much stigma combined with ABI, acceptance of being a person with an ABI can be difficult and extremely shameful. We need to validate this fact much more in rehabilitation and in society. Moreover, rethink why professionals think insight is so important? Could it not serve a protective function when clients challenge the ABI? Maybe we should start validating and normalizing accounts from ABI survivors as good practice instead of confrontation and correction of reality, since this traditional practice seams to fail its purpose of leading to more insight and acceptance of difficulties and thus to a greater motivation to work with these difficulties. Furthermore, we should start reflecting on what happens to an individual's identity when they are met with disbelief in what they are saying. We should create more sameness with ABI survivors, not difference. This approach contrasts a more traditional and distanced professional/expert vs. client approach and underscores the need for a new knowledge regime in neurorehabilitation.

What seems to make the crucial difference in rehabilitation is “the way of being” of the professional. The importance of an emotional connection and of understanding the other as a complete person was underpinned by Carl Rogers, founder of humanistic psychology, years ago, but have we forgotten this valuable insight into relations? Within neurorehabilitation most educations and courses for professional focus on how we can increase our neurological knowledge on brain injuries. Rarely there are courses focusing on the relationship between professionals and adults with ABIs. Therefore, it seems important to revitalize some of Rogers valuable insight when humans are dealing with humans.

Regarding the BackUp-manual the therapist noted that the content and exercises of session number 9 could be moved forward. This would be in line with the therapy progression described in CFT (53).

### Limitations and Future Directions for Studies in the Area

People with ABI represent a very heterogenous group, and thus many factors we cannot control for (31). Therefore, case studies are considered very appropriate to evaluate interventions (57). However, case studies hold some limitations, including lack of generalizability. The purpose of this study was not to produce generalizable findings, but to make a first evaluation of the BackUp-manual used as an intervention program in a single case study, and how the existing rehabilitation could benefit from a psychological intervention. The BackUp-program has not been thoroughly evaluated before and given the benefits of single case study to explore a new intervention program, the case study was assessed to be the most suited for the purpose (31). However, it is important to stress that more cases is needed to validate the effect.

The assessment tests did not show clear effect of the intervention, but the evaluating interview did. However, Lars' experienced problems remembering the intervention at 9 months follow-up, which highlights the need for follow up interviews to be conducted right after the intervention. Another factor that can affect the outcome, is that the participant in this case study tends to be motivated by participating in the study (31).

Based on the findings and limitations of this study it needs to be evaluated if inclusions criteria need to be set in regard to initial scores on the outcome measurement, or if other outcome measures are needed for future studies. The outcome measures used in this study are based on previous studies in this field (24). However, the manual was originally designed to support the psychological rehabilitation after an ABI; therefore, more qualitative measurements about obtaining a therapy goal or questionnaires on treatment satisfaction after every session could be relevant. Another assessment tool that could be suggested to befit and supplement a qualitative evaluation of the feasibility of the intervention, could be a standardized questionnaire for the therapist, evaluating the client's commitment to the therapy and the feasibility of the sessions.

As put forward by Gracey et al. (57), we recommend further case studies of psychological intervention programs as part of a holistic rehabilitation program following ABI. Future case studies should enroll more cases. Additionally, future studies should explore the link between different interventions that are part of a holistic rehabilitation in order to better assess the interrelation of various interventions.

## Conclusion

Overall, this case study provides support for a psychological intervention based on the BackUp-manual, specifically designed to support the psychological rehabilitation after an ABI. Further studies are needed to explore the benefits of a psychological interventions as a part of a holistic rehabilitation program after acquired brain injury.

## Data Availability Statement

The original contributions presented in the study are included in the article/supplementary material, further inquiries can be directed to the corresponding author/s.

## Ethics Statement

The studies involving human participants were reviewed and approved by the Danish Data Protection Agency (Datatilsynet). The project was also reported to the Regional Research Ethics Committees for the Region of Nothern Jutland (Nordjylland) who found it exempt from full review. The patients/participants provided their written informed consent to participate in this study.

## Author Contributions

All authors agreed to be accountable for the content of the work. All authors contributed to the article and approved the submitted version.

## Conflict of Interest

The authors declare that the research was conducted in the absence of any commercial or financial relationships that could be construed as a potential conflict of interest.

## Publisher's Note

All claims expressed in this article are solely those of the authors and do not necessarily represent those of their affiliated organizations, or those of the publisher, the editors and the reviewers. Any product that may be evaluated in this article, or claim that may be made by its manufacturer, is not guaranteed or endorsed by the publisher.
